# Survival of porcine epidemic diarrhea virus (PEDV) in thermally treated feed ingredients and on surfaces

**DOI:** 10.1186/s40813-017-0064-3

**Published:** 2017-09-19

**Authors:** Michaela P. Trudeau, Harsha Verma, Pedro E. Urriola, Fernando Sampedro, Gerald C. Shurson, Sagar M. Goyal

**Affiliations:** 1 Department of Animal Science, University of Minnesota, 1988 Fitch Ave, Falcon Heights, MN 55108 USA; 2 Veterinary Population Medicine, University of Minnesota, 1365 Gortner Avenue, St. Paul, MN 55108 USA

**Keywords:** Thermal processing, Feed ingredients, Porcine epidemic diarrhea virus, Inactivation, Survival, Surfaces

## Abstract

**Background:**

Infection with Porcine Epidemic Diarrhea Virus (PEDV) causes vomiting, diarrhea, and dehydration in young pigs. The virus made its first appearance in the U.S. in 2013, where it caused substantial neonatal mortality and economic losses in the U.S. pork industry. Based on outbreak investigations, it is hypothesized that the virus could be transmitted through contaminated feed or contaminated feed surfaces. This potential risk created a demand for research on the inactivation kinetics of PEDV in different environments. Therefore, the objective of this study was to evaluate the survival of PEDV in 9 different feed ingredients when exposed to 60, 70, 80, and 90 °C, as well as the survival on four different surfaces (galvanized steel, stainless steel, aluminum, and plastic).

**Results:**

Overall, there were no differences (*P* > 0.05) in virus survival among the different feed matrices studied when thermally processed at 60 to 90 °C for 5, 10, 15, or 30 min. However, the time necessary to achieve a one log reduction in virus concentration was less (*P* < 0.05) when ingredients were exposed to temperatures from 70 °C (3.7 min), 80 °C (2.4 min), and 90 °C (2.3 min) compared with 60 °C (4.4 min). The maximum inactivation level (3.9 log) was achieved when heating all ingredients at 90 °C for 30 min. There were no differences in the amount of time necessary to cause a one log reduction in PEDV concentration among the different surfaces.

**Conclusions:**

The results of this study showed that PEDV survival among the 9 feed ingredients evaluated was not different when exposed to thermal treatments for up to 30 min. However, different combinations of temperature and time resulted in achieving a 3 to 4 log reduction of PEDV in all feed ingredients evaluated. Finally, PEDV survival was similar on galvanized steel, stainless steel, aluminum and plastic.

## Background

Upon infection with Porcine Epidemic Diarrhea Virus (PEDV), pigs experience vomiting, diarrhea, and dehydration leading to high mortality in suckling pigs [1]. The virus is excreted in large amounts in the feces of infected pigs, making it highly contagious and difficult to control [2]. After the virus was identified in Belgium in 1978, it slowly spread to multiple countries including Canada, Korea, and China [3]. In the United States, the virus was first detected in May of 2013, and while the mode of introduction has not yet been confirmed, contaminated feed has been suspected as the cause of transmission [4].

Recent research on PEDV survival in feed ingredients has shown that it appears to survive longer in soybean meal (greater than 180 days) compared with other commonly used feed ingredients [5]. The authors also showed that PEDV can survive for up to 30 days in blood meal, corn dried distiller’s grains with solubles, meat and bone meal, red blood cells, L-lysine HCl, D, L-methionine, choice white grease, choline chloride, and complete feed. However, the research determined virus survival when samples were stored at low, uncontrolled temperatures (varying between −15 to 20 °C) and did not investigate the impact of any thermal processing treatment. Results from other studies suggest that specific thermal processing treatments, such as spray drying (a process using dry hot air to reduce the moisture of a particle) can reduce the survival of PEDV in porcine and bovine plasma by 5 log [6, 7]. Other research has shown that conditioning and pelleting with temperatures above 54.4 °C could be effective in reducing infectivity of PEDV in swine feed [8]. However, the sole impact of thermal processing on the inactivation kinetics of PEDV in feed is still unknown.

If a feed ingredient is contaminated when it enters the feed mill, it has been shown to contaminate the feed mill surfaces [9]. Previous research has shown differences in the effectiveness of decontamination treatments between various equipment and facility surface materials including metal, plastic, rubber, and concrete [10]. This surface contamination with PEDV can then contaminate subsequent batches of feed [11]. If this type of contamination occurs, it is necessary to understand virus inactivation kinetics on different surface materials before a treatment is applied. However, the survival of PEDV on various surfaces is not well known.

The objectives of this study were to measure the effect of thermal treatment on inactivation kinetics of PEDV in nine commonly used feed ingredients, and to determine the PEDV inactivation kinetics on various equipment and facility surfaces (i.e. galvanized steel, stainless steel, aluminum, and plastic). We hypothesized that different chemical characteristics of the feed ingredients would affect PEDV survivability when subjected to different thermal treatments and PED virus inactivation would differ among material surfaces.

## Methods

### Virus propagation

The NVSL strain of PEDV was grown in Vero-81 cells, which were grown in Dulbecco’s Modified Eagle Medium (Mediatech, Herndon, VA), 8% fetal bovine serum (FBS; HyClone, South Logan, UT), 50 μg/mL gentamicin (Mediatech, Herndon, VA), 150 μg/mL neomycin sulfate (Sigma, St. Louis, MO), 1.5 μg/mL fungizone (Sigma, St. Louis, MO), and 455 μg/mL streptomycin (Sigma, St. Louis, MO). Before inoculation, the cells were washed 3 times with phosphate buffered Saline solution (pH 7.2). After inoculation, the cells were incubated at 37 °C allowing virus absorption using maintenance medium (DMEM, antibiotics, and 10.0 μg/mL trypsin; Gibco, Life Technologies, Grand Island, NY). After 1 h, new media were added to the flask and the cells were placed in an incubator at 37 °C under 5% CO_2_. The cells were examined daily for the appearance of cytopathic effects (CPE), usually appearing 4 to 5 days post-infection. After CPE was observed, the cells underwent 3 freeze-thaw cycles (−80 °C to 25 °C) and were then centrifuged at 2500×g for 15 min at 4 °C. After centrifugation, the supernatant was collected, aliquoted in 25 mL tubes, and stored at −80 °C until used.

### Feed ingredients composition

Feed ingredients (i.e. soybean meal, swine growing-finishing vitamin and trace mineral premix, spray dried porcine plasma, meat meal, meat and bone meal, blood meal, corn, and corn distillers dried grains with solubles) were obtained from the feed mill at the Southern Research and Outreach Center of the University of Minnesota (Waseca, MN). The sample of complete feed evaluated was a phase II starter diet that did not contain any animal by-products (Vita-Plus CGI, enhanced NP-NT, batch no. 831458). All feed and feed ingredients were tested and confirmed negative for PEDV by real time RT-PCR. Samples were sent to Minnesota Valley Testing Laboratory (New Ulm, MN) to analyze the nutrient composition of each ingredient (Table 1). Standard procedures established by AOAC International were used to measure moisture (method 930.15), ash (method 942.05), ether extract (method 2003.05), crude fiber (method 930.39), and crude protein (method 990.03) content [12]. The proximate analysis values were obtained from a single sample. The pH was measured by mixing 50 mL of distilled water with 5 g of each feed ingredient, premix, and complete feed. The mixture was then stirred with a magnetic stirrer for 20 min. The pH of the suspended feed was measured using a pH probe (Fisher Scientific, Waltham, MA) and recorded. All chemical composition values for each ingredient were determined from one replicate. The pH of each sample was measured in triplicate.

**Table 1:** Chemical composition of common feed ingredients used in diets for pigs

Ingredient^a^	Moisture (%)	Ash (%)	Ether extract (%)	Crude fiber (%)	Crude Protein^b^ (%)	pH^c^
CF	8.57	9.45	4.47	2.02	24.20	5.82
SBM	12.12	6.42	0.71	3.26	45.40	6.73
C	14.90	1.55	3.86	1.55	7.03	6.21
DDGS	10.31	4.56	5.86	6.50	30.10	4.39
PM	2.41	73.77	1.42	1.62	1.91	3.49
SDPP	11.60	7.44	0.15	< 0.01	77.79	7.15
BM	11.58	1.79	0.16	0.05	92.60	8.40
MM	4.80	24.26	13.54	1.83	54.90	6.64
MBM	5.74	24.77	10.77	1.16	55.70	6.50

^a^ *CF* complete feed, *SDPP* spray dried porcine plasma, *MM* meat meal, *MBM* meat and bone meal, *BM* blood meal, *SBM* soybean meal, *C* corn, *PM* vitamin-trace mineral premix, *DDGS* Corn distillers dried grains with solubles

^b^Crude protein is calculated from nitrogen content × 6.25

^c^Average of 3 replicates

### Virus survival in feed ingredients after thermal processing

Five gram aliquots of each ingredient were weighed into plastic scintillation vials (Fisher Scientific, Pittsburgh, PA) and placed into sealed, airtight, and water proof containers. Preliminary experiments measured the temperature of the feed and feed ingredients after being placed in the water bath and determined that 1 h was required for feed to achieve the maximum temperature of the water bath. During the experiment, the containers were placed in a water bath at 60, 70, 80 and 90 °C for 1 h to reach water bath temperature. Once the samples reached the desired temperature, they were removed and 1 mL of PEDV (passage 19, titer 3.2 × 10^4^ TCID_50_/mL) was added to the samples. During this time, the samples were removed from the water bath for about 5 min to complete the inoculation procedure. The inoculated samples were then immediately placed back into the water bath for 0, 5, 10, 15, or 30 min.

To elute the surviving virus from the samples of feed and feed ingredients, an eluent solution, 3% beef extract (Lab Scientific, Highlands, NJ) 0.05 M glycine (Sigma), pH 7.2 was used. After various time points, this solution was added to the sample aliquot and mixed well. After light centrifugation to remove organic debris, the supernatant was collected. To determine the concentration of surviving virus, a titration was performed by preparing serial 10-fold dilutions of the supernatant in maintenance medium. These dilutions were inoculated into monolayers of Vero-81 cells grown in 96 microtiter well plates (Nunc, Rochester, NY) at 100 μL/well using three wells per dilution. The inoculated cells were incubated at 37 °C under 5% CO_2_ for 4 to 5 days and observed for CPE. The virus titer was then calculated as 50% tissue culture infective dose (TCID_50_/mL) [13]. The virus titers of the supernatants were compared to those of the initial virus titer to determine the amount of virus inactivation.

### Virus survival in equipment surfaces

A total of 4 surface materials were evaluated including stainless steel, aluminum, plastic, and galvanized steel. Stainless steel and aluminum sheets were purchased from Hardware Hank (St. Paul, MN). For the plastic surface, 6-well plastic plates (Nunc, New York, NY) were used, and galvanized steel (28 gal. Silver Galvanized Steel Hobby Sheet Sleeved; Model # 57321) was obtained from Home Depot (Roseville, MN).

The PEDV inoculation solution (40 μL) was applied to the center of a sample of stainless steel, aluminum, plastic, or galvanized steel. The virus was allowed to dry for 10 min, and the sheets and plates were then stored at room temperature (~25 °C) for up to 10 days. At 0, 1, 2, 5, and 10 days, surviving virus was eluted from the center of a surface using 400 μL of an elution buffer (3% beef extract in 0.05 M glycine, pH 7.2). To elute the virus, the elution buffer was applied to the surface and then removed off the surface with a pipette. At the time 0 elution point, 78% of the virus was recovered using this method. After elution, the sample was titrated in Vero-81 cells to determine virus concentration. This experiment was then repeated once more to provide a total of two experiments.

### Calculations and statistical analyses

Inactivation kinetics data on virus survival were analyzed using the Weibull model [14]. The fitting of the model to the experimental data was performed by using the GINAFiT add-in software on Microsoft excel [15]. Assuming that the temperature resistance for PEDV follows a Weibull distribution, an equation was used to predict the log concentration of surviving virus after the thermal treatment (Eq. 1):

1 $$ Log(N)= Log\left({N}_0\right)-{\left(\raisebox{1ex}{$ t$}\!\left/ \!\raisebox{-1ex}{$\delta $}\right.\right)}^n $$

In eq. 1, *N* is the surviving virus expressed as TCID_50_/mL, *N*_*0*_ is the initial virus titer at the start of the experiment, *t* is time (min), *δ* is the time of the first log reduction of virus concentration (min), and *n* is the shape parameter. The shape parameter (*n*) indicates the shape of the curve with a value *n* > 1 representing the formation of a shoulder-shaped curve and being convex, and *n* < 1 represented the formation of a tail-shaped curve and concave in shape, while *n* = 1 represented a linear function. The adjusted R^2^ value (Adj. R^2^) was used to evaluate how well the model fit the experimental data.

The delta values obtained from the Weibull model indicated the amount of time necessary to reduce the virus concentration by 1 log. The delta values were compared across treatments. Normality was assessed using the UNIVARIATE procedure of SAS. An ANOVA statistical analysis using the PROC-MIXED procedure of SAS was performed to determine statistically significant differences between feed ingredients and between temperature treatments. When evaluating virus survival between the four temperatures, feed ingredient was considered a random effect. Least squared means with a Tukey adjustment were used to determine differences among each treatment if *P* < 0.05. The experimental unit was a single vial.

## Results

There were no differences (*P* > 0.05) in survival of PEDV after the thermal treatment among the 9 feed materials evaluated (Table 2). This observation was consistent at each of the 4 temperatures applied, indicating that the virus resistance to thermal treatment was not affected by the different chemical composition of the feed matrices. Delta values at 70 to 90 °C were less (*P* < 0.05) than at 60 °C (Table 3), indicating higher virus inactivation kinetics at greater temperatures. The shape parameter of the virus inactivation curves were not different among treatments, and values ranged between 0.45 and 0.60, indicating that at each temperature, curves were concave and formed tails. This behavior corresponds to a rapid decrease of virus concentration after short treatment times followed by a plateau where the virus survived for an extended period of time.

**Table 2:** Weibull model kinetic parameters of Porcine Epidemic Diarrhea Virus survival in ingredients after thermal treatment

Temperature	60 °C		70 °C		80 °C		90 °C	
Ingredient^a^	Delta (min)^b^	Adj. R^2^	Delta (min)^b^	Adj. R^2^	Delta (min)^b^	Adj. R^2^	Delta (min)^b^	Adj. R^2^
CF	3.8 ± 1.2	0.72	1.1 ± 1.3	0.88	1.5 ± 1.6	0.84	2.0 ± 2.2	0.84
SBM	3.3 ± 2.3	0.83	1.3 ± 5.0	0.83	1.7 ± 1.8	0.68	2.0 ± 2.1	0.85
C	3.4 ± 3.2	0.85	3.3 ± 4.5	0.75	2.2 ± 1.5	0.90	1.7 ± 1.6	0.89
DDGS	2.5 ± 1.7	0.84	2.2 ± 2.4	0.87	1.3 ± 1.4	0.87	2.1 ± 1.7	0.87
PM	4.9 ± 4.3	0.89	1.4 ± 5.0	0.76	2.0 ± 2.4	0.85	2.0 ± 1.7	0.83
SDPP	3.6 ± 3.4	0.86	2.1 ± 1.8	0.86	2.3 ± 1.6	0.85	2.1 ± 3.1	0.87
BM	2.0 ± 6.0	0.83	3.5 ± 4.4	0.81	1.5 ± 2.8	0.84	0.64 ± 0.3	0.84
MM	3.0 ± 2.6	0.85	2.0 ± 1.6	0.89	2.1 ± 1.2	0.90	2.1 ± 0.9	0.84
MBM	6.0 ± 2.5	0.88	2.4 ± 1.4	0.84	2.3 ± 1.0	0.85	0.9 ± 0.9	0.75
*P*-Value	0.75		0.50		0.98		0.78	

^a^ *CF* complete feed, *SDPP* spray dried porcine plasma, *MM* meat meal, *MBM* meat and bone meal, *BM* blood meal, *SBM* soybean meal, *C* corn, *PM* vitamin-trace mineral premix, *DDGS* Corn distillers dried grains with solubles

^b^Average of 6 replicates, Delta values indicates the time to achieve 1 log reduction

**Table 3:** Survival of Porcine Epidemic Diarrhea virus (PEDV) in feed and feed ingredients when thermally treated

Temperature	Average *δ* ^1,2^ (min)	Shape Paremeter^3^	Adj. R^2^	Log reduction at 10 min^1^	Log reduction at 15 min^1^	Log reduction at 30 min^1^
60 °C	4.4^a^ ± 3.5	0.50	0.83	1.7^a^ ± 0.4	2.0^a^ ± 0.4	2.4^a^ ± 0.4
70 °C	3.7^b^ ± 3.7	0.45	0.84	1.9^a^ ± 0.5	2.3^a^ ± 0.4	2.7^a^ ± 0.7
80 °C	2.4^b^ ± 1.8	0.50	0.85	2.2^b^ ± 0.3	2.8^b^ ± 0.8	3.4^b^ ± 0.9
90 °C	2.3^b^ ± 1.9	0.60	0.84	2.6^c^ ± 0.9	3.3^c^ ± 1.1	3.9^c^ ± 0.8
*P*-Value	0.0002			0.0001	0.0001	0.0001

^1^Different letters in the same column differ at *P* < 0.05

^2^ *δ* is the time of the first log reduction of virus concentration

^3^The shape parameter (*n*) indicates the shape of the curve with a value *n* > 1 forming shoulders and being convex, *n* < 1 forming tails and being concave, and *n* = 1 being linear

When comparing the log reduction achieved after 10, 15, or 30 min, no differences (*P* > 0.05) were observed when the virus was exposed to 60 and 70 °C, but greater reductions (*P* < 0.05) in virus concentration were achieved at 80 and 90 °C. A reduction of 1.9 to 2.0 log was achieved at 60 °C for 15 min, or 70 °C for 10 min, and a 2.2 to 2.4 log reduction occurred after treatment at 60 °C for 30 min, 70 °C for 15 min, or 80 °C for 10 min. Greater than a 3 log reduction was observed when applying 80 °C for 30 min (3.4 log), or 90 °C for 15 min (3.3 log). The maximum log reduction (3.9 log) was achieved at 90 °C for 30 min.

During the 10-day incubation period, PEDV titer was reduced by 1.3 log on all surfaces except for stainless steel, in which only a 0.83 log reduction was observed. The PEDV remained viable (10^2^ TCID_50_/g) on each of the four surfaces after 10 days of incubation. When delta values were compared, there were no differences among all 4 surfaces ranging from 0.7 and 7.7 days (Table 4).

**Table 4:** Concentration of viable Porcine Epidemic Diarrhea virus (PEDV) after inoculation in various surfaces

	Concentration of viable PEDV (Log TCID_50_/mL) on:			
Time (days)	Stainless steel	Aluminum	Plastic	Galvanized steel
0	3.51	3.51	3.51	3.51
1	2.51	2.51	2.51	2.51
2	2.51	2.51	2.51	2.51
5	2.45	1.70	1.51	2.51
10	2.70	2.18	2.18	2.18
Weibull model				
Delta, days	7.72 ± 7.16	0.79 ± 0.10	0.69 ± 0.00	1.74 ± 0.00
Adjusted R^2^	0.88	0.67	0.56	0.94
Delta *P-*value	> 0.05			

## Discussion

Recent investigations have shown that feed contaminated with PEDV is capable of infecting pigs [16]. Therefore, it is important to develop mitigation strategies to reduce the risk of virus transmission to swine farms through contaminated feed. Previous research has suggested that contaminated feed ingredients can be a risk factor for PEDV transmission among swine farms, and that virus survival was different among ingredients [5]. Varying PEDV survival among feed ingredients suggests that feed ingredients may need to be handled and processed differently based on virus inactivation kinetics and relative risk of transmission for a specific feed ingredient. When thermal treatment of complete feed was evaluated at high temperatures, heating complete feed at 120 °C for 25 min resulted in a 3 log reduction in PEDV [17]. However, the previous study was performed using complete feed, and there has been limited information published regarding thermal treatment of PEDV in individual feed ingredients. If a feed ingredient is contaminated, studies have shown that it can then contaminate surfaces in a feed mill [9]. After surface contamination with PEDV, subsequent batches of feed can be contaminated with the virus [11]. Therefore, it is necessary to determine if any differences in virus survival among feed ingredients requires different thermal processing conditions to reduce the risk of subsequent contamination. Additionally, it is necessary to understand the inactivation kinetics of PEDV on various surfaces of materials used in feed mills and swine farms.

Our hypothesis was that PEDV survives differently in complete feed or feed ingredient varying in chemical composition, and that some ingredients may require greater processing temperatures to achieve an adequate virus inactivation. Our study evaluated feed ingredients, premix, and complete feed with different chemical composition and pH values. However, no differences were observed among the virus inactivation kinetics (delta values). These results suggest that under the conditions evaluated in this study (high temperatures and long exposure times), rapid virus inactivation may occur independently of chemical composition of ingredients, and thus, similar processing conditions can be applied to all ingredients to achieve a similar reduction in virus concentration. Our results were unexpected because of the dramatic differences in the pH values of the feed matrices evaluated (3.49 to 8.40). Quist-Rybachuk et al. (2015) found that PEDV was more heat sensitive when the pH increased from 7.2 to 10.2 [18]. The lack of differences in PEDV inactivation among ingredients despite the pH differences may also be due to the use of dry ingredients instead of liquid media. Because pH is only a characteristic of solutions, the impact of the pH on virus survival in a dry ingredient with a small amount of liquid (1 mL) is likely to be minimal. In addition to this, the maximum pH in our experiment was only 8.40, which is considerably lower than the pH of 10.2 that created variation in virus sensitivity to thermal treatments in previous experiments [18].

Although there were no differences in virus survival among the feed materials evaluated between 60 and 70 °C, greater virus inactivation was achieved at 80 and 90 °C. In order to optimize the thermal processing conditions (high temperature and short time) to inactivate PEDV, our data suggest that thermal treatment at 80 °C for 15 min was necessary for achieving a 3-log reduction. This extent of inactivation could also be achieved by thermal processing at 90 °C for 10 min or heating at 70 °C for 30 min. These findings and parameters are consistent with those reported by Hoffman and Wyler (1989), who found that PEDV was relatively stable at 50 °C, but at temperatures greater than 60 °C, the virus lost total infectivity within 30 min [19]. These results were also comparable to the survival of PEDV on the metal surface of hog transport trailers, where heating at 71 °C for 10 min was capable of reducing virus titer low enough to not cause infection in any of the 4 inoculated pigs, however, the exact reduction of PEDV was not measured [20].

If a contaminated ingredient enters the feed mill, it has been demonstrated that this ingredient will contaminate feed mill surfaces and subsequent batches of feed [9]. This research has evaluated the contamination of feed mill surfaces, but limited studies have been conducted on PEDV long-term survival after a surface is contaminated. Data from this experiment suggest that PEDV can survive for extended periods of time on all of the material surfaces evaluated, which are in agreement with other reports in the literature. In similar experiments, Casanova et al. (2010) found that TGEV, another swine coronavirus, can remain infectious on hard non-porous surfaces for up to 28 days [21]. In that study, there was a 3.2 log reduction in TGEV after 28 days at room temperature at 80% relative humidity. Another human coronavirus, SARS, has been actively studied for its survival on different material surfaces [21]. This virus has been reported to survive for up to 36 h on stainless steel, but the initial concentration of virus in this study was not reported [22]. In a different experiment, Rabenau et al. (2005) reported a 4-log reduction in SARS virus concentration after 9 days of incubation on a polystyrene surface [23]. Furthermore, SARS virus survived on smooth plastic more than 5 days at room temperature [24]. Results from these previous studies, along with similar examples [25, 26], indicate that coronaviruses may pose a risk for transmission via contaminated surfaces in the feed mill. Our results showed longer virus survival time (greater than 10 days), which indicates that additional mitigation measures (i.e. proper cleaning and disinfection) need to be implemented to minimize risk of virus transmission on surfaces of feed mills and swine farms.

One of the limitations for applying this combined knowledge into practice is the potential experimental methodology concern of adding 1 mL of media containing the virus to feed samples. The addition of liquid media necessarily increases the moisture content of the sample, and this may affect the virus survival. More research is necessary to compare the effect of moisture content and water activity on PEDV survival, and determine the extent that this factor plays in virus inactivation. In the surface experiment, however, the media was allowed to dry, eliminating this factor as a potential limitation. It is highly likely that the amount of virus excreted by an infected pig, and potentially transmitted via feed, would be much greater than the titer used in the present study. In a study that evaluated residual material in a suspected PEDV contaminated feed bin, CT values between 19.5 and 22.2 were determined [16]. When using a calibration curve obtained from the University of Minnesota and published by Alonso et al. (2014), this amount of virus is equivalent to 8.9 to 9.2 log copies of RNA/g [27]. In this potential scenario, the maximum log reduction (3.9 log) achieved by thermal processing alone, would not be enough to completely inactivate the virus found in the feces of infected animals, and would have the potential to be transmitted via feed during a PEDV outbreak. If this scenario represents the reality, a new approach is needed that is able to achieve a greater reduction. A hurdle approach (combining multiple processing steps) may be needed to achieve the desired virus reduction. The use of eBeam irradiation, antimicrobials, and organic acids has been effective in reducing PEDV concentration in feed [17]. If these treatments are combined with a thermal processing as described in this study, an overall increase on virus inactivation will be expected.

## Conclusions

Complete feed, vitamin-trace mineral premix, and feed ingredients are potential biosecurity risk factors in the widespread of PEDV to pork production facilities around the world if they become contaminated. The results of this study indicate that there are no differences in virus survival among complete feed, premix, and ingredients with different chemical composition when thermally treated at temperatures greater than 70 °C, suggesting that similar processing conditions will be effective to inactivate PEDV across all types of feed materials. A maximum of 4-log reduction was achieved when applying 90 °C for 30 min. PEDV inactivation kinetics (delta values) did not differ among surfaces tested, which indicated that all surfaces have the same relative risk of PEDV transmission.

## Abbreviations

CPE: Cytopathic effects
DMEM: Dulbecco’s Modified Eagle Medium
PEDV: Porcine Epidemic Diarrhea Virus
SDPP: Spray dried porcine plasma
TCID_50_: Median Tissue Culture Infectious Dose

## References

[1] Stevenson GW, Hoang H, Schwartz KJ, Burrough EB, Sun D, Madson D (2013). Emergence of porcine epidemic diarrhea virus in the United States: clinical signs, lesions, and viral genomic sequences. J Vet Diagn Investig.

[2] Pensaert MB, de Bouck P (1978). A new coronavirus-like particle associated with diarrhea in swine. Arch Virol.

[3] Song D, Park B (2012). Porcine epidemic diarrhoea virus: a comprehensive review of molecular epidemiology, diagnosis, and vaccines. Virus Genes.

[4] Bowman AS, Krogwold RA, Price T, Davis M, Moeller SJ (2015). Investigating the introduction of porcine epidemic diarrhea virus into an Ohio swine operation. BMC Vet Res.

[5] Dee S, Neill C, Clement T, Singrey A, Christopher-hennings J, Nelson E. An evaluation of porcine epidemic diarrhea virus survival in individual feed ingredients in the presence or absence of a liquid antimicrobial. Porc Heal Manag. 2015:1–10. doi:10.1186/s40813-015-0003-0. 10.1186/s40813-015-0003-0 PMC5382523 28405416

[6] Gerber PF, Xiao CT, Chen Q, Zhang J, Halbur PG, Opriessnig T (2014). The spray-drying process is sufficient to inactivate infectious porcine epidemic diarrhea virus in plasma. Vet Microbiol.

[7] Pujols J, Segales J (2014). Survivability of porcine epidemic diarrhea virus (PEDV) in bovine plasma submitted to spray drying processing and held at different time by temperature storage conditions. Vet Microbiol.

[8] Cochrane RA, Schumacher LL, Dritz SS, Woodworth JC (2017). Effect of pelleting on survival of porcine epidemic diarrhea virus – contaminated feed 1. J Anim Sci.

[9] Schumacher LL, Cochrane RA, Evans CE, Kalivoda JR, Woodworth JC, Stark CR (2015). Evaluating the effect of manufacturing Porcine Epidemic Diarrhea Virus (PEDV) -contaminated feed on subsequent feed mill environmental surface contamination evaluating the effect of manufacturing porcine epidemic diarrhea virus. Kansas Agric Exp Stn Res Rep..

[10] Huss AR, Schumacher LL, Cochrane RA, Poulsen E, Bai J, Woodworth JC (2017). Elimination of porcine epidemic diarrhea virus in an animal feed manufacturing facility. PLoS One.

[11] Schumacher LL, Cochrane RA, Woodworth JC, Stark CR (2015). Utilizing feed sequencing to decrease the risk of Porcine Epidemic Diarrhea Virus ( PEDV ) cross- contamination during feed manufacturing utilizing feed sequencing to decrease the risk of porcine epidemic. Kansas Agric Exp Stn Res Rep.

[12] Horwitz W, Latimer GW (2005). Official methods of analysis of AOAC international.

[13] Karber G (1931). Fifty percent endpoint calculation. Arch Exp Path Pharmak.

[14] Mafart P, Couvert O, Gaillard S, Leguerinel I (2001). On calculating sterility in thermal preservation methods : application of the Weibull frequency distribution model. Acta Hortic.

[15] Geeraerd AH, Valdramidis VP, Van Impe JF (2005). GInaFiT, a freeware tool to assess non-log-linear microbial survivor curves. Int J Food Microbiol.

[16] Dee S, Clement T, Schelkopf A, Nerem J, Knudsen D, Christopher-Hennings J (2014). An evaluation of contaminated complete feed as a vehicle for porcine epidemic diarrhea virus infection of naive pigs following consumption via natural feeding behavior: proof of concept. BMC Vet Res.

[17] Trudeau MP, Verma H, Sampedro F, Urriola PE, Shurson GC, McKelvey J (2016). Comparison of thermal and non-thermal processing of swine feed and the use of selected feed additives on inactivation of Porcine Epidemic Diarrhea Virus (PEDV). PLoS One.

[18] Quist-rybachuk GV, Nauwynck HJ, Kalmar ID (2015). Sensitivity of porcine epidemic diarrhea virus ( PEDV ) to pH and heat treatment in the presence or absence of porcine plasma. Vet Microbiol.

[19] Hofmann M, Wyler R (1989). Quantitation, biological and physicochemical properties of cell culture-adapted porcine epidemic diarrhea coronavirus (PEDV). Vet Microbiol.

[20] Thomas P, Karriker LA, Ramirez A, Zhang J, Ellingson JS, Holtkamp DJ (2014). Methods for inactivating PEDV in hog trailers. Twenty-second annual swine disease conference for swine Parctitioners.

[21] Casanova LM, Jeon S, Rutala WA, Weber DJ, Sobsey MD (2010). Effects of air temperature and relative humidity on coronavirus survival on surfaces. Appl Environ Microbiol.

[22] Organization WH, Organization WH (2003). First data on stability and resistance of SARS coronavirus compiled by members of WHO laboratory network.

[23] Rabenau HF, Cinatl J, Morgenstern B, Bauer G, Preiser W, Doerr HW (2005). Stability and inactivation of SARS coronavirus. Med Microbiol Immunol.

[24] Chan KH, Peiris JS, Lam SY, Poon LL, Yuen KY, Seto WH (2011). The effects of temperature and relative humidity on the viability of the SARS coronavirus. Adv Virol.

[25] Bean B, Moore BM, Sterner B, Peterson LR, Gerding DN, Balfour HH (1982). Survival of influenza viruses on environmental surfaces. J Infect Dis.

[26] Blachere FM, Lindsley WG, Pearce TA, Anderson SE, Fisher M, Khakoo R (2009). Measurement of airborne influenza virus in a hospital emergency department. Clin Infect Dis.

[27] Alonso C, Goede DP, Morrison RB, Davies PR, Rovira A, Marthaler DG (2014). Evidence of infectivity of airborne porcine epidemic diarrhea virus and detection of airborne viral RNA at long distances from infected herds. Vet Res.

